# Effects of adding psychosocial stimulation for children of lactating mothers using an unconditional cash transfer platform on neurocognitive behavior of children in rural Bangladesh: protocol for a cluster randomized controlled trial

**DOI:** 10.1186/s40359-019-0289-9

**Published:** 2019-03-05

**Authors:** Sheikh Jamal Hossain, Bharati Rani Roy, Nur-E Salveen, Mohammad Imrul Hasan, S. M. Mulk Uddin Tipu, Shamima Shiraji, Fahmida Tofail, Jena D. Hamadani

**Affiliations:** Maternal and Child Health Division (MCHD), Internationational Centre for Diarrhoeal Disease Research, Bangladesh (icddr,b), Mohakhali, Dhaka, 1212 Bangladesh

**Keywords:** Bangladesh, Behavior, Cognitive, Development, Language, Motor, Psychosocial stimulation, Unconditional cash transfer

## Abstract

**Background:**

There is sufficient evidence that psychosocial stimulation (PS) benefits children’s neurocognitive behavior, however, there is no information on how it works when delivered through an Unconditional Cash Transfer (UCT) platform for poor rural population in developing countries. The objective of this study is to measure effects of adding PS for children of lactating mothers enrolled to receive UCT with health education (HE) on neurocognitive behavior of children in rural Bangladesh.

**Methods:**

The study will be conducted at 11 unions of Ullapara sub-district in Bangladesh. The study is a cluster randomized controlled trial with three-arms; (i) PS and UCT with HE (ii) UCT with HE and iii) Comparison arm. The cluster will be considered as an old *Ward* of a *Union*, the lowest tier of local government system in rural Bangladesh. There are three old *Wards* in a *union*. These three clusters will be randomized to one of the three arms. Similarly, randomization will be done for each 11 Unions and then 11 clusters will be assigned to an arm. Eighteen participants will be recruited from each cluster randomly (*n* = 196 in each arm). The intervention designed for one year includes UCT with HE for the poor as a safety net program in rural Bangladesh with or without PS. An age-based curriculum of PS is already available for Bangladeshi children and this will be administered by trained local women; play leaders (PL) in intervention clusters. The government of Bangladesh is providing UCT of taka 500 ($6.25) as maternity allowance per month with HE. The primary outcomes will be cognitive, motor and language composite scores measured by Bayley-III and behavior using Wolke’s behavior rating scale. The secondary outcomes will be children and mothers’ growth, family food security status, health seeking behavior, mothers’ depressive symptoms and self-esteem and violence against mothers.

**Discussion:**

The study will provide a unique opportunity to assess an integrated early childhood development intervention using UCT platform to mitigate developmental delays in poor vulnerable children of rural Bangladesh.

**Trial registration number:**

ClinicalTrials.gov NCT03281980, registered on September 13, 2017.

## Background

An estimated 250 million children below 5 years in low and middle income countries do not reach their maximum potential because of poverty and other risk factors e.g. malnutrition, stimulation, postpartum depression etc. [1]. Sometimes poor people become poorer due to catastrophic expenditure on health care and fall into the vicious cycle of poverty [2]. Social safety net program is a way of achieving wellbeing for the vulnerable. Conditional and unconditional cash transfer to the poor mothers has proved a way of reducing poverty and improving health outcomes for mothers and children worldwide [3–6]. Conditional cash transfer (CCT) alone was also found significantly effective for child development e.g. Oportunidades/Progresa program in Mexico [6]. On the other hand, studies reported that unconditional cash transfer (UCT) improved health outcomes [7] and nutrition [8]. The latest Lancet series on child development documented social safety net programs e.g. conditional and unconditional cash transfer, a suitable means for improving children’s development [9]. There is little information about effect of UCT alone on children’s development. Psychosocial stimulation alone and along with other health and nutrition programs was found to be effective for children’s cognition and behavior [10–14].

Ministry of Women and Children Affairs (MOWCA), Government of Bangladesh (GOB) provides maternity allowance for rural poor mothers; an unconditional cash transfer under safety net program of GOB. The mothers also receive limited health education (HE) program. The overall objective of the cash and health education (UCT-HE) training program is to ensure safety net in terms of morbidity, mortality and welfare during pregnancy and lactation period for both mother and child. Adding psychosocial stimulation to the program is proven to benefit children’s cognition, behavior and nutritional status. On the other hand the interventions together will address mothers’ physical and mental health and self-esteem and family’s food security because receiving extra cash as well as education on health and psychosocial stimulation will enhance mothers’ capacity in many aspects.

But little is known about the effects of psychosocial stimulation and UCT with HE program on children’s cognition and behavior.

## Methods

### Research objectives

The primary objective of this study is to measure the effects of adding psychosocial stimulation to an UCT-HE program on cognitive, language and motor development and behavior of young children. The secondary objectives are to measure effects of the programs on:

- Children’s growth
- Mothers’ nutritional status, mental health (depression symptoms), self-esteem and exposure to domestic violence
- Household food security status, health seeking behavior and health care expenditure
- To evaluate cost effectiveness of the intervention

### Hypothesis

Our primary hypotheses are: 1) UCT-HE program will improve children’s cognitive, motor and language development and behavior compared to comparison group; 2) Adding psychosocial stimulation to UCT-HE program will have an additive effect on children’s cognitive, motor and language development and behavior compared to the UCT-HE group.

Our secondary hypotheses are that additionally the intervention will: 1) improve children and mothers’ nutritional status; 2) reduce mothers’ depressive symptoms and improve their self-esteem and reduce exposure to domestic violence; 3) improve household food security status, health seeking behavior and health care expenditure and 4) be cost effective.

### Study area

The study area is Ullapara, Sirajgonj, a sub-district of rural Bangladesh. There are 14 *Unions* in this sub-district. Like many other sub-districts risk factors for poor development e.g. poverty, malnutrition and unstimulating home environment, are all prevalent in the study area. Rural areas are much alike in terms of demography, topography, economy and health system in Bangladesh.

### Study design

This is a cluster randomized controlled trial with three arms: i) Psychosocial stimulation and UCT-HE ii) UCT-HE and iii) Comparison arms. The comparison arm will consist of those who are eligible to receive UCT but do not receive it due to resource constraints of the government.

### Sample size

The sample size is estimated based on an effect size of 0.45 SD and considering 80% power, 5% level of significance, and intraclass correlation (ICC) of 0.05 and an attrition rate of 25%.. In our previous studies of psychosocial stimulation alone, we have achieved an effect size of 0.52 SD on children’s development [15], but here we use effect size of 0.45 SD to be conservative. The study therefore requires 594 mother-child dyads in 33 clusters with 18 participants in each cluster.

### Intervention

The intervention includes:

i) Psychosocial stimulation and
ii) UCT (Maternity allowance) with HE awareness program

Psychosocial stimulation: The participants will receive fortnightly sessions of psychosocial stimulation at home by community female play leaders for one year. The play leaders will be selected from the same locality and will be trained on the curriculum of psychosocial stimulation for 2 weeks. They will visit children’s homes carrying home-made toys and picture books and will demonstrate play activities to the mothers. The toys will be left in the homes at each visit and exchanged with new toys on the following visit. The play leaders will also convey messages on language development and nutritional care for the children. The curriculum was first developed and used in Jamaica and then a culturally modified and translated version was developed for use in Bangladesh [16]. The curriculum is based on improving the mother-child interaction and providing developmentally appropriate activities for the child. The curriculum has already been used in Bangladesh by Child Development Unit of icddr,b and has shown significant benefits to children’s development [11–13, 15] and growth [12, 15].

Unconditional Cash Transfer (Maternity allowance) with Health education awareness program: MOWCA provides maternity allowance of taka 500 ($6.25) for each targeted poor pregnant mother under safety net program of the GOB. The mothers receive the cash every six months for two years through banking channel. The objectives of the allowance are to help improving mothers and children’s health and wellbeing. The eligibility criteria for getting UCT and HE are:

1) mother’s age is equal to or more than 20 years; 2) mother is pregnant during UCT enrolment 3) the mother has no child or one child; 4) monthly income of the mother is less than Taka 1500; 5) the family owns a residence only or lives in residence of others; 6) the mother or the family do not have agricultural land or pond for fish culture and 7) poor and disabled women will have priority to receive UCT .

All mothers in each *union* under the UCT (maternity allowance) program will receive training by designated local NGOs (LNGOs) or community based organizations (CBOs) through MOWCA, GOB funding. HE programme is designed to educate mothers on theirs and their children’s health and nutrition, women’s right and welfare in a broad way. The mothers are also motivated to spend the allowance for improving their and their child’s health and welfare. MOWCA developed and approved the curriculum for HE program, which the mothers are supposed to receive in 13 sessions over two years of the UCT program. The number of eligible mothers to receive UCT with HE is larger than those who receive it, because of resource constraints of GOB.

### Participants recruitment and randomization

Eleven *Unions* will be randomly selected out of 14 *Unions* from Ullapara, Sirajgonj sub-district in rural Bangladesh. A *Union* is the lowest tier of local government system in the country. Three old *Wards* together make a *Union*. On an average there are 10 thousand populations in an old *Ward*. Many administrative and resource allocation are done considering the old *Wards* as a small unit in the health system of Bangladesh. This old *Ward* will be considered as a cluster for the study. A restricted randomization process will be followed where each of the three clusters of a *Union* will be randomly allocated to one of the three arms. Therefore, 11 *Wards* will fall under each of the three arms.

The participants will be the recipients of UCT -HE program or those eligible for the program but not receiving it. Since the Government is providing UCT -HE to 79 mothers in each *Union,* the research team is expecting to get approximately 26 participants in each cluster. On an average18 participants will be recruited randomly in each cluster of the arm i) and ii).

**Fig. 1:**
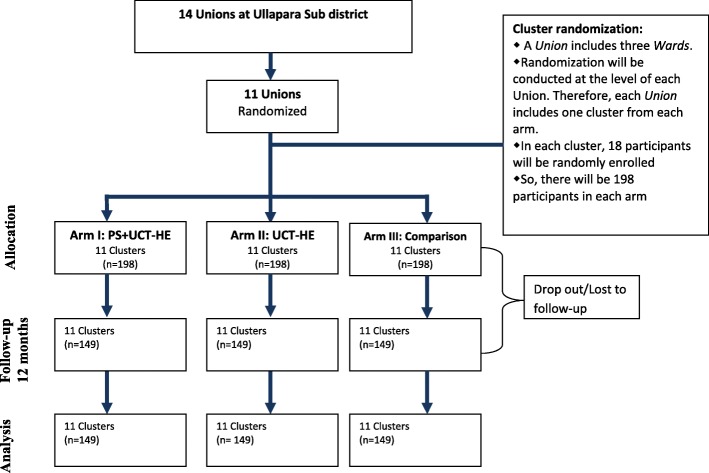
Flow chart of the cluster randomization controlled trial

We will collect a list to identify potential beneficiaries from the clusters of the comparison arm (arm iii) through survey following the eligibility criteria of the recipients for the UCT - HE program. Then we will exclude the recipients of the UCT -HE program from the list through discussion with local government authority of Ullapara sub district. Finally, we will enrol 18 participants randomly in each cluster of this arm (arm iii). We will not impede providing GOB program in the comparison clusters due to ethical consideration and it is a limitation for the study. Permission from the GOB official will be obtained to use the information about the participants. The recruitment and randomization process has been shown in the trial flow chart (Fig. 1).

### Eligibility criteria for the study

Participants will be assigned to the study only if they meet all of the inclusion criteria and none of the exclusion criteria.

### Inclusion criteria

- Mothers with a child aged 6–16 months
- Eligible to receive UCT.
  - ○ Psychosocial stimulation and UCT with HE arm: Receiving unconditional cash from government
  - ○ UCT with HE arm: Receiving unconditional cash from government
  - ○ Comparison arm: eligible to receive UCT but not under UCT program.
- Not expected to leave the study site for more than 2 months
- Has a legally acceptable representative capable of understanding the informed consent document and providing consent on the participant’s behalf.

### Exclusion criteria

- Legal guardian unwilling or unable to provide written informed consent.
- Known congenital anomaly, developmental disorder or severe developmental delay
- If the child cannot be tested due to physical or behavioral problems
- Children of multiple birth e.g. twin, triplets

### Training, data collection and data quality

All testers will be trained intensively for one month on Bayley Scales of Infant and Toddler Development (third edition), Wolke’s behavior ratings, anthropometry and other data collection tools. When the inter observer reliability coefficient will be more than 0.8, the trainee will be considered to be eligible to take part in the test procedure. Quality of data collection will be checked at the field by master trainers on 10% of the assessments. The testers will be blinded to the intervention status of the participants. The outcomes and measurement tools and time line of measurements are shown in the table. (Table 1).

**Table 1:** Descriptions of measurement of outcomes/variables and its time line

Outcomes/variables	Tools.	Descriptions	Time
Children’s cognitive, language and motor development	Bayley III	This tool was adapted and used in a number of studies by Chid Development unit, icddr,b [18].	Baseline and end line
Children’s behavior	Wolk’s behavior rating scale	This tool has been used in a number of studies by Chid Development unit, icddr,b [19] .	Baseline and end line
Children’s growth	WHO growth standards	Children’s length/height, weight, Occipitofrontal circumference and Mid Upper arm circumference (MUAC) based on WHO guidelines	Baseline and end line
Mothers’ depressive symptoms	Self Reporting Questionnaire (SRQ-20)	SRQ-20 questionnaire has been used in our settings and the result has also been published in peer reviewed journal [20]	Baseline and end line
Mother’s self-esteem	Rosenberg’s Self-Esteem questionnaire	This scale has been validated (Illyas 2003 unpublished data) and used in our settings [21]	Baseline and end line
Domestic violence	Pretested Questionnaire	Type of violence against mother, e.g. physical mental, perpetrator, etc.	Monthly
Household food security status	Household Food Insecurity Access Scale (HFIAS)	This questionnaire has been used in our settings [18]	Monthly
Health seeking behavior and health care expenditure	Pretested questionnaire	Health seeking behavior, e.g. type of health problem, providers, and facilities, and cost of health care etc.	Monthly
Family Care Indicators	Validated questionnaire	Child development care and practice information. This tools was validated by our group [22]	Baseline and end line
Monthly income and expenditure	Pretested questionnaire	Sources of income and expenditure	Monthly
Mothers’ anthropometry	WHO standard	Mothers’ height, weight and MUAC	Baseline and end line
Socioeconomic data	Pretested questionnaire	Socioeconomic background of the participants	Baseline and end line
Cost data	Pretested questionnaire	Direct and indirect cost	Last quarter of the study and at the end

Play leaders assigned for psychosocial stimulation will be trained on the curriculum for 2 weeks and will be monitored by the supervisors. Weekly trouble shooting meetings will be held, any serious problem will be discussed and measures will be taken to solve it. The play leaders will receive monthly one-day refresher training on psychosocial stimulation. The investigators with long experience in the field of public health research will ensure smooth running of project activities in every step.

### Data storage and record retention

The data will be password protected and only members of the study team and investigators will have access to the password. Only unidentified data will be stored on the database.

Data will be available to the principal investigator. Following the archival period, hard copies of the data will be destroyed according to local procedures.

### Data analysis

All questionnaires will be checked and cross checked and necessary corrections will be made before data entry. Pre-coded questionnaire will be used to minimize data coding errors. Data will be checked for normality before analysis. Appropriate measures will be taken if there are any abnormal distributions. Potential confounding variables and effect modifiers will be considered for all analyses.

Background information will be presented in tabular forms with number and percentage in the three groups. Differences between groups in socioeconomic and other background characteristics will be analyzed using t-test for continuous variables for two groups and ANOVA for three groups. Intention-to-treat analysis controlling for the clustering effect will be followed using multiple regression analysis.

### Cost-effectiveness analysis

The objective of this analysis is to estimate the resource use and costs associated with interventions. We will measure direct and indirect intervention cost. Average costs per participants will be calculated by multiplying the cost of resource items by their respective unit costs. The analysis will be from the societal perspective. We will also measure the outcomes/effectiveness of the study. Then the incremental cost-effectiveness ratio (ICER) will be used in cost-effectiveness analysis.

### Data sharing

Data will be publicly available in an accessible format as per icddr,b data policy.

### Trial status

Ongoing.

## Discussion

Prior to designing the study, we were looking for a platform to reach the poor population with this intervention in the country. But there are very few suitable platforms to deliver child development package for the children under three years of age. The health system of Bangladesh is not sufficient to address developmental delay for all the poor children in the country because of many supply and demand barriers. We aim to cover those poor children using UCT platform. We are assuming that there will be an additive or synergistic effect because of integration of psychosocial stimulation and UCT. So a positive finding of this study will pave the way for a new platform for an integrated UCT and psychosocial stimulation service delivery to prevent developmental delay of poor rural children.

The first aim and objective of ‘the National Children Policy 2011’ is to ensure optimum child development and growth of the children in the country irrespective of any economic, social, gender and geographical barriers [17]. Moreover, MOWCA, the concern ministry for early child development activity in the country, approved Comprehensive Early Childhood Care and Development Policy in 2013 covering conception to 8 years as early child developmental period for the first time. But most of those programs target children over three years and little is going on for the age group conception to three years. Our integrated intervention will be an opportunity to document findings in low resource settings for the age group below three years. A negative finding of this study would be used to inform research agenda.

## Abbreviations

CBOs: Community based organizations
CCT: Conditional cash transfer
GOB: Government of Bangladesh
HE: Health Education
ICER: Incremental cost effectiveness ratio
MOWCA: Ministry of Women and Children Affairs
NGOs: Non government organizations
PL: Play leader
PS: Psychosocial stimulation
SD: Standard deviation
UCT: Unconditional cash transfer
